# In vitro investigations on interference of selected probiotic candidates with *Campylobacter jejuni* adhesion and invasion of primary chicken derived cecal and Caco-2 cells

**DOI:** 10.1186/s13099-024-00623-x

**Published:** 2024-06-21

**Authors:** Thomas Willer, Zifeng Han, Colin Pielsticker, Silke Rautenschlein

**Affiliations:** Clinic for Poultry, University of Veterinary Medicine Hannover, Foundation, Buenteweg 17, 30559 Hanover, Germany

**Keywords:** *Campylobacter jejuni*, Primary chicken intestinal cells, Probiotics, Colonization, Cytokines

## Abstract

**Background:**

*Campylobacter (C.) jejuni* is one of the most important bacterial foodborne pathogens worldwide. Probiotics such as *Lactobacillus* or *Bacillus* species are considered one option for reducing the colonization rate and magnitude in poultry, the most frequent source of human infections. Due to the lack of suitable avian in vitro models such as chicken intestinal cell lines, especially those derived from the cecum, most in vitro studies on *C. jejuni* host interaction have been conducted with human intestinal cell lines. In this study, we compared *C. jejuni*-cell interactions between primary chicken cecal cells and the human intestinal cell line Caco-2, which is derived from colorectal adenocarcinoma, and investigated possible interfering effects of selected probiotic candidates.

**Results:**

We detected differences in adhesion and invasion between the two tested gut cell types and between different *C. jejuni* strains. The probiotic inhibition of *C. jejuni* adhesion and invasion of human and avian gut cells was affected by host cell type, investigated *C. jejuni* strain and time points of probiotic treatment. Additionally, our results suggest a possible correlation between *C. jejuni* invasion and the detected increase in IL-6 mRNA expression.

**Conclusions:**

Our results indicate distinct differences between avian and human gut cells in their interaction with *C. jejuni.* Therefore, data obtained in one host species on *C. jejuni*-host interaction may not easily be transferrable to another one. The factors influencing the variable efficacy of probiotic intervention in chicken and human derived cells should be investigated further.

## Background

Campylobacteriosis is one of the most widespread infectious gastrointestinal disease worldwide with an increasing incidence not only in developing but also in industrialized countries. It may be considered as endemic in some regions in the world, specifically in young children and young adults [1]. Since 2005, Campylobacteriosis has been recognized as the major bacterial foodborne disease in the European Union [2]. The infection of humans with *Campylobacter (C.). jejuni* normally manifests as self-limiting diarrhea. But there is also a risk of the development of complications such as the Guillain–Barré and Miller Fisher syndrome [3]. The consumption of contaminated chicken meat is currently the most common way of infection for humans [2, 4]. Therefore, chickens are considered the most important reservoir for *C. jejuni*.

*C. jejuni* was classified for decades as a commensal of the chicken and was not further investigated with respect to *C. jejuni*-host interactions. Currently, there is increasing evidence that *C. jejuni* may also be a pathogen for chickens [5] and may lead to pathological disorders under certain circumstances. This was reviewed in detail by Awad et al. [6].

*C. jejuni*-host interaction in poultry has only been insufficiently investigated so far. However, for the implementation of more sufficient control strategies, this relationship has to be elucidated further [7]. Most investigations on the interaction of *C. jejuni* with its host have been performed in vitro using human-derived cell lines such as HEp-2, INT407 or Caco-2 [8, 9]. However, more recent studies have shown differences in *C. jejuni* colonization ability and proinflammatory responses between human- and nonhuman-derived cells [10, 11] and even between different human intestinal cell lines [12–14].

The lack of chicken cell lines of intestinal origin limits the number of studies on the interaction of *C. jejuni* with avian host cells under controlled conditions [10, 15–19]. Recently, an embryo-derived avian cell line from the duodenum was developed, but no cell line from the cecum, the location with the highest *C. jejuni* colonization load, of posthatch birds has been established [20].

According to a report released by the European Food Safety Authority (EFSA), a reduction in *C. jejuni* colonization of the intestine by 3 log_10_ units at slaughter would reduce the public health risk by at least 90% [21]. Thus, methods to reduce the *C. jejuni* burden at the flock level, such as vaccination and pro- or prebiotic administration, are needed but have not been successfully established in the field yet with repeatable reduction rates.

The goal of this study was to obtain deeper insights into the host-*C. jejuni* interactions using not only human but also avian-derived intestinal cells. We compared the adhesion and invasion rates of Caco-2 cells derived from a human colorectal adenocarcinoma [22] with those of primary chicken intestinal epithelial cells (CIECs). Furthermore, *C. jejuni* isolates of different origins were selected, and their in vitro colonization patterns and the expression of selected proinflammatory cytokines were more closely investigated in CIECs. In addition, the influence of three selected probiotics on the colonization of CIECs by *C. jejuni* was investigated and compared to that of the human intestinal Caco-2 cell line.

## Results

### Investigations of the interaction between *C. jejuni* and primary CIECs

**Fig. 1:**
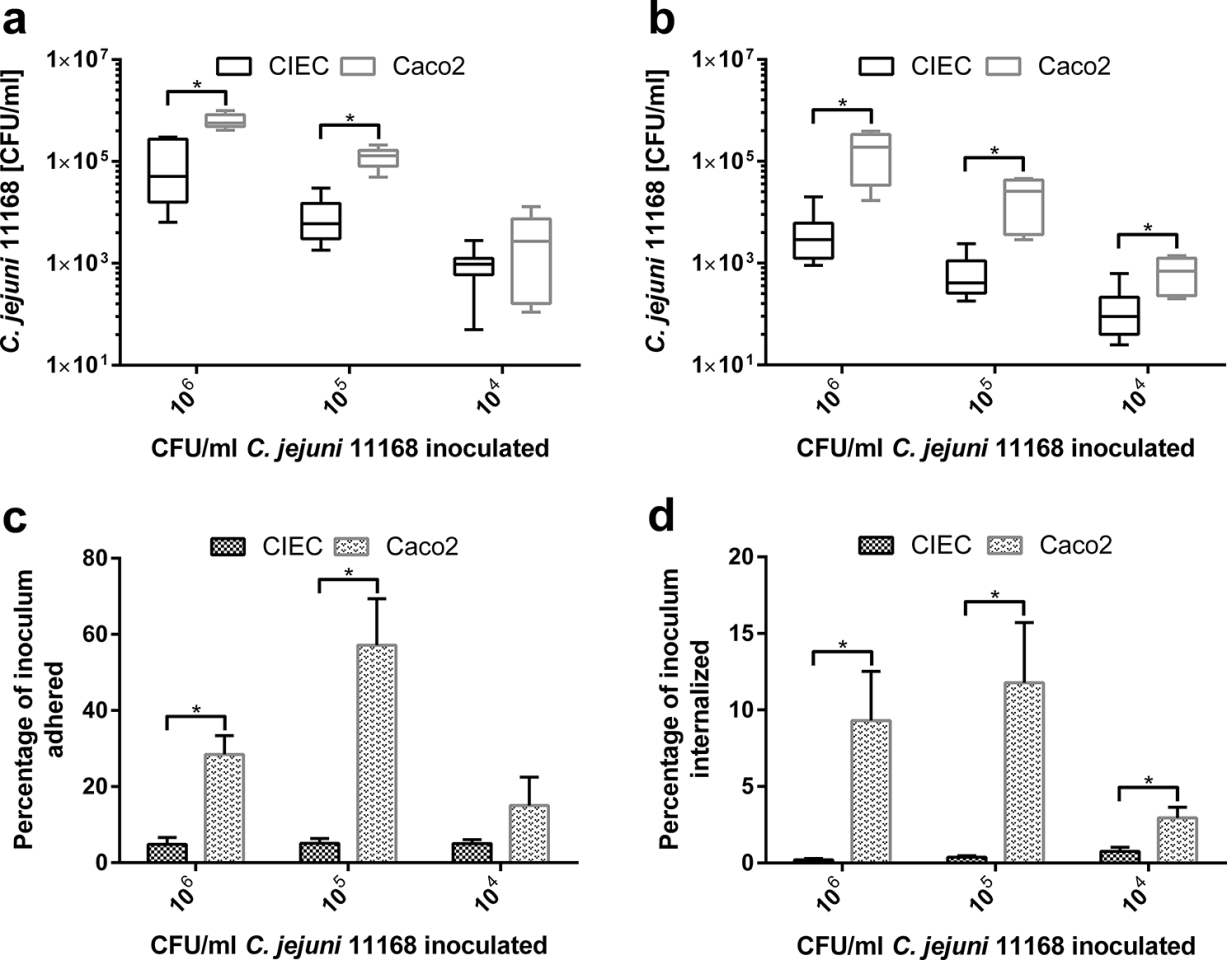
Adhesion and invasion of CIECs and Caco2 after inoculation with *C. jejuni* strain 11168. Absolute adhesion (**a**), absolute invasion (**b**), relative adhesion (**c**) and relative invasion (**d**) investigated three hours after inoculation of CIEC and Caco2 cells with 10^4^, 10^5^ and 10^6^ CFU/ml *C. jejuni* 11168. The results are presented as CFU/ml cell lysate (absolute adhesion and invasion) and as percentage of the inoculum adhered/internalized (relative adhesion and invasion). The presented data are the means of two (Caco2) or three (CIEC) independent experiments with pools of different donor chickens performed in triplicate. Error bars indicate the standard error of the mean (SEM). (*p* < 0.05; two-sample t test, Wilcoxon rank sum test)

Adherence and invasion are important virulence mechanisms for pathogenic bacteria. Therefore, we compared the adherence and invasion of avian CIECs and human Caco-2 cells treated with different doses of 10^4^-10^6^ colony forming units (CFU)/mL *C. jejuni* reference strain 11168. *C. jejuni* adherence to Caco-2 cells was approximately one log greater than that to CIECs, which was statistically significant when *C. jejuni* was inoculated at doses of 10^5^ and 10^6^ CFU/ml (*p* < 0.01). *C. jejuni* invasion exhibited a similar pattern to that of adhesion, with a significantly greater invasion rate in Caco-2 cells than in CIECs at all the tested *C. jejuni* inoculation doses (*p* < 0.05; Fig. 1a + b, Experiment 1).

When the numbers of adherent and invading bacteria were related to the number of CFU in the inoculum (Fig. 1c + d), only 4.8–5.1% and 0.2–0.8% of the inoculum were detected in association with the CIECs, respectively, while the CFU were greater (*p* < 0.05) and more variable for Caco2 cells depending on the number of inoculated bacteria.

**Fig. 2:**
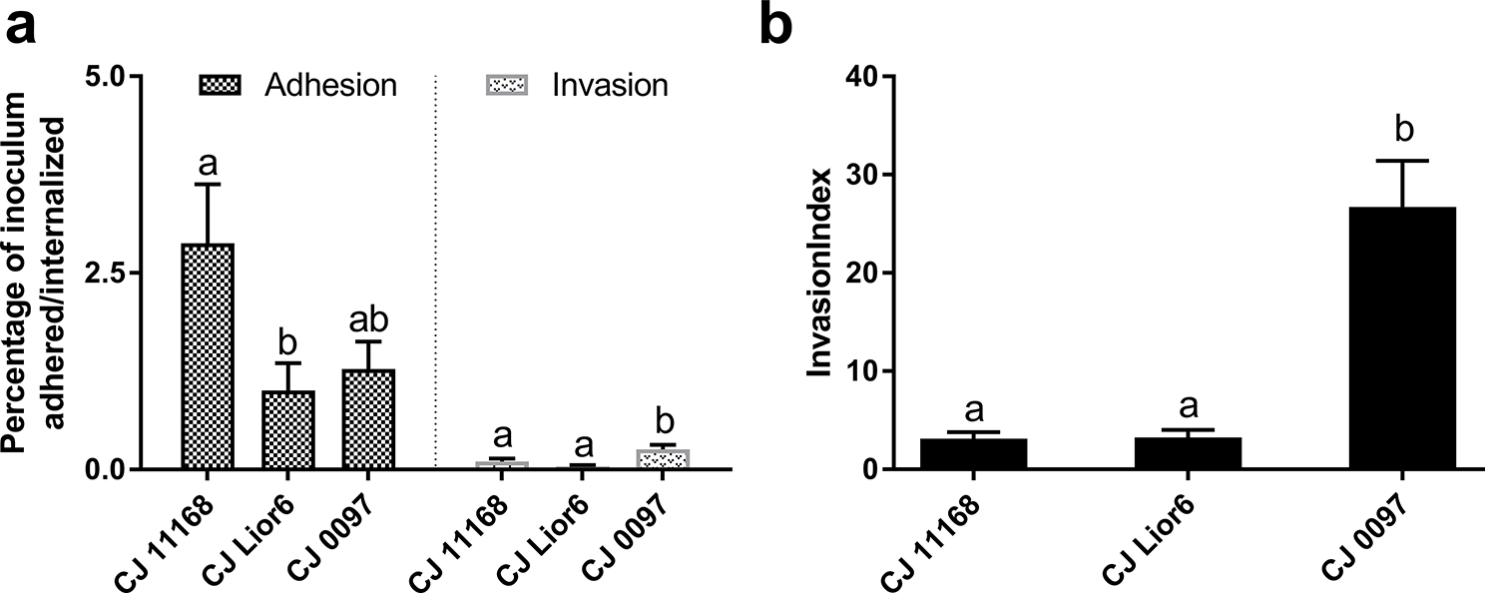
Colonization patterns after inoculation of CIECs with three different *C. jejuni* strains. Relative adhesion and invasion (**a**) and invasion indices (**b**) after inoculation of CIECs with 10^6^ CFU/ml of one of the three different *C. jejuni* strains (11168, Lior6, or 0097). The results are presented as the percentage of CFU of *C. jejuni* in the inoculum that adhered or were internalized (relative adhesion and invasion) and the percentage of total CFU of cell-associated *C. jejuni* that were internalized (invasion index). The presented data are the means of three independent experiments with pools of different donor chickens performed in triplicate. Error bars indicate the standard error of the mean (SEM). Letters indicate significant differences between strains (*p* < 0.05; one-way analysis of variance with Tukey’s HSD All-Pairwise Comparisons Test)

The colonization patterns of the three different *C. jejuni* strains 11168, Lior6 and 0097 were compared on CIEC to investigate the specific effects of the strains (Experiment 2). We detected an increase in the adhesion rate of 11168 that was more than twofold greater than that of Lior6 or 0097. In contrast, 0097 had the significantly highest invasion activity on CIECs, while the invasion of Lior6 and 11168 was low. This resulted in a high invasion index, the percentage of adhered *C. jejuni* internalized, of 26.7% for 0097 and low invasion indices of 3.1 for 11168 and 3.3% for Lior6 (Fig. 2).

After infection of CIECs with 10^6^ CFU/ml *C. jejuni* 11168, Lior6 or 0097, the expression patterns of the interleukin (IL)-1β and IL-6 mRNAs, which are proinflammatory cytokines known to be upregulated after *C. jejuni* infection of chickens [23, 24], were investigated via qRT‒PCR. There were no clear differences in the mRNA expression of IL-1β or IL-6 after *C. jejuni* inoculation after eight hours post inoculation (hpi) in a preliminary experiment. Therefore, we limited the investigation of cytokine expression to four and eight hours after inoculation.

**Fig. 3:**
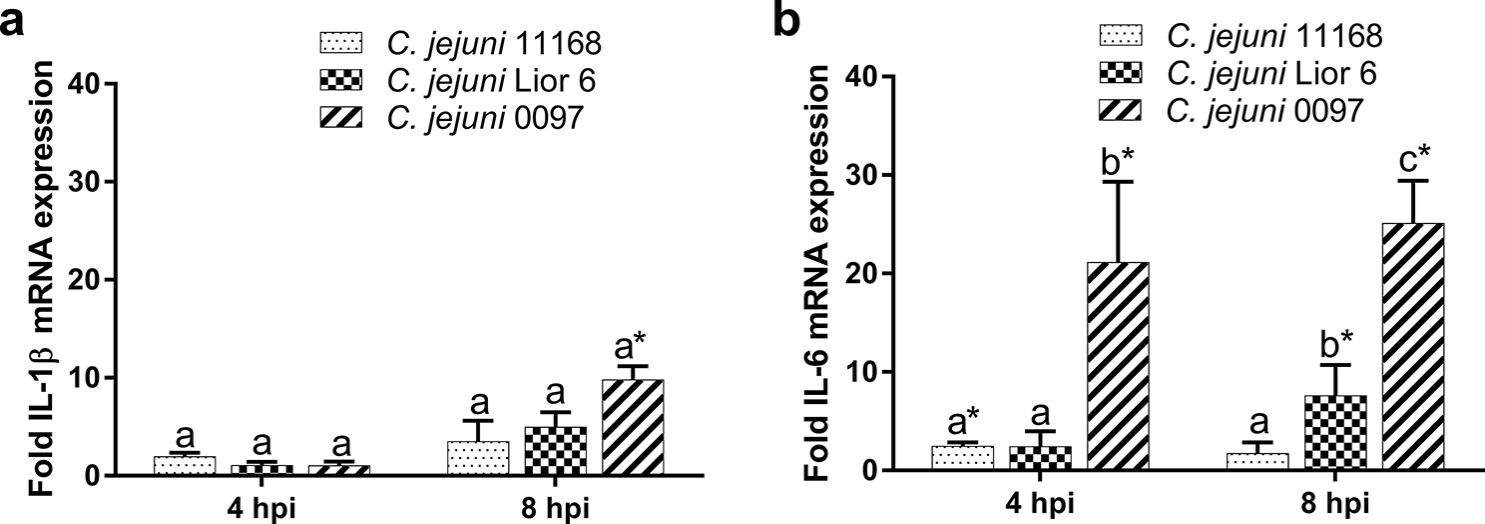
Interleukin (IL)-1β and IL-6 mRNA expression in CIECs after inoculation with *C. jejuni*. CIECs were inoculated with 10^6^ CFU/ml of one of three different *C. jejuni* strains (11168, Lior6, or 0097). Cells were collected four and eight hours post inoculation, and IL-1β (**a**) and IL-6 (**b**) mRNA expression was investigated. The results are presented as the fold change in expression compared to that in noninoculated control cells. Relative quantification was performed by qRT‒PCR, and expression values were normalized to 28 S rRNA. Error bars indicate the standard error of the mean (SEM). Letters indicate significant differences between strains. (*n* = 5–7 replicates; *p* < 0.05; one-way analysis of variance with Tukey’s HSD All-Pairwise Comparisons Test). Asterisks indicate significant differences compared to the noninoculated controls at the investigated time points (*p* < 0.05; two-sample t test)

Overall, the expression level of IL-1β mRNA was low in the *C. jejuni*-inoculated and *C. jejuni*-free groups, in which the values ranged from 5.6 to 7.1 ΔC_t_-40. Only *C. jejuni* 0097 induced a statistically significant upregulation of IL-1β mRNA expression at eight hpi compared to that in the non-inoculated controls (*p* < 0.05). There was no statistically significant difference in the IL-1β mRNA level between cells inoculated with either one of the three *C. jejuni* strains at any time point (*p* > 0.05). The expression of IL-6 mRNA was clearly upregulated after *C. jejuni* 0097 inoculation at four and eight hpi compared to that in the noninoculated controls (*p* < 0.01), while Lior6 induced a statistically significant but distinct decrease in the upregulation of IL-6 mRNA expression at eight hpi (*p* < 0.01). IL-6 expression in CIECs was slightly but significantly upregulated after *C. jejuni* 11168 inoculation at four hpi (*p* < 0.01; Fig. 3).

### Investigation of direct and indirect probiotic effects

**Fig. 4:**
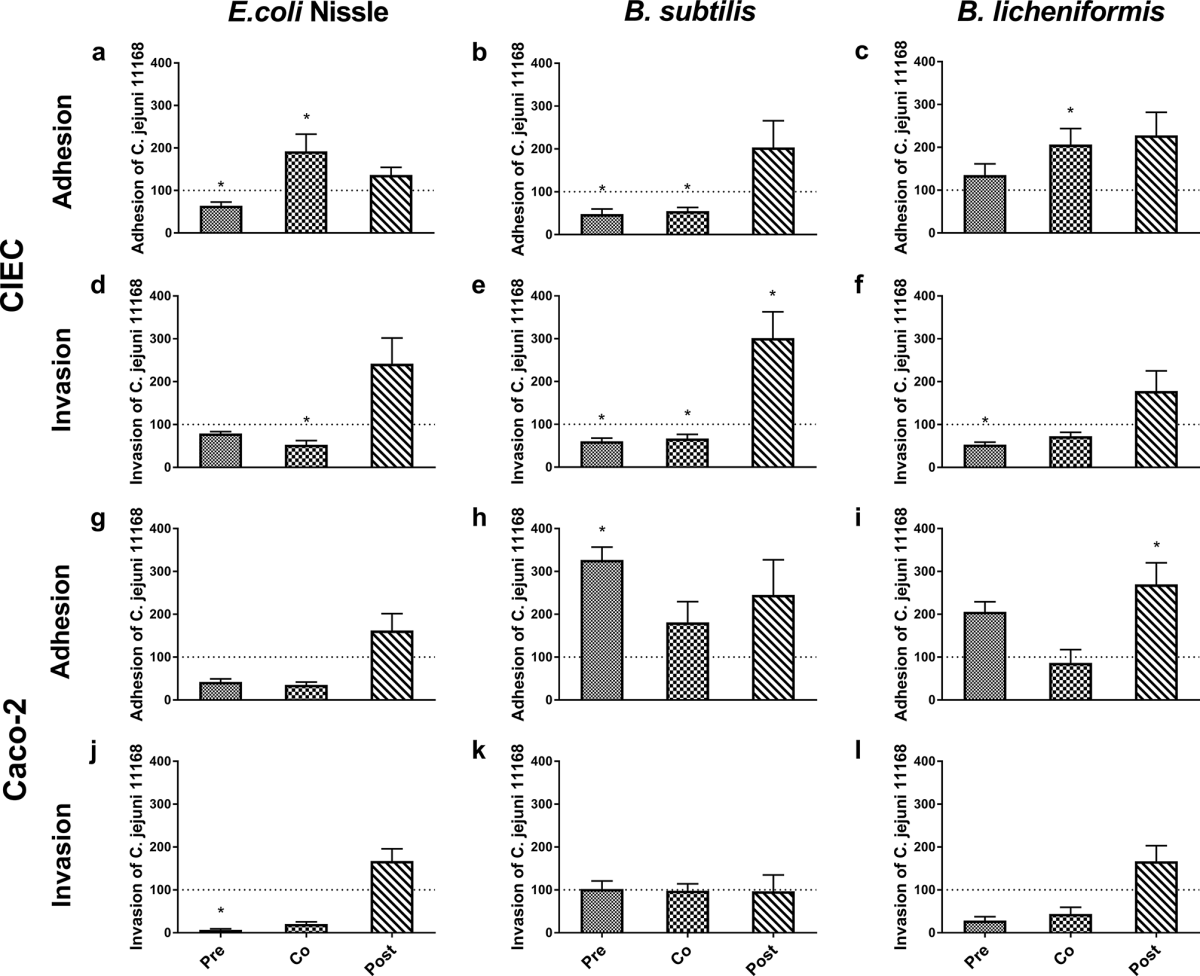
Influence of incubation time point of probiotics and *C. jejuni* on colonization by *C. jejuni* 11168. CIEC (**a**-**f**) and Caco-2 (**g**-**l**) cells were inoculated with 10^6^ CFU/ml *C. jejuni* 11168 either after 1 h or 1 h before inoculation with the probiotic *E. coli* NISSLE (**a**, **d**, **g**, **j**; inoculation dose 10^6^ CFU/ml), *B. subtilis* DSM 17299 (**b**, **e**, **h**, k; inoculation dose 10^5^ CFU/ml) or *B. licheniformis* DSM 17236 (**c**, **f**, **i**, **l**; inoculation dose 10^6^ CFU/ml). The results are presented as the percentage of adherent (a-c and g-i) or invasive (**d**-**f** and **j**-**l**) cells compared to that of the *C. jejuni* monoinoculated cells (100%, as marked by the dotted line) in the same experiment. Pre = preincubated; Co = coincubated; Post = postincubated. The presented data are the means of two independent experiments performed in triplicate with pooled CIECs from different donor chickens. Error bars indicate the standard error of the mean (SEM). Asterisks indicate significant differences compared to the noninoculated control at three hours post *C. jejuni* inoculation (*p* < 0.05; two-sample t test, Wilcoxon rank sum test)

None of the six tested culture supernatants of the probiotic *candidates—Escherichia coli* NISSLE (EcN), *Bacillus subtilis* DSM 17299 (BS), *Bacillus licheniformis* DSM 17236 (BL), *Clostridium butyricum* DSM 10702 (CB), *Enterococcus faecium* DSM 7134 (EF), or *Lactobacillus rhamnosus* DSM 7133 (LR)—had inhibitory effects on any of the three *C. jejuni* strains according to the Agar Well Diffusion Assay (data not shown). *Escherichia coli* Nissle (EcN), *Bacillus subtilis* (BS) and *Bacillus licheniformis* (BL), which showed promising results in their probiotic effects on *C. jejuni* according to preliminary tests, were selected and tested for their ability to interfere with the adhesion and invasion of *C. jejuni* 11168 on CIEC (Fig. 4a-f). Investigations were also performed on Caco-2 cells (Fig. 4g-l) to determine the effects of the species and cell line and for better comparability to the literature with respect to the different genetic and morphological backgrounds of the applied cell lines. When added and incubated one hour after *C. jejuni* inoculation (post-incubation) none of the probiotics inhibited adhesion or invasion of *C. jejuni*. In most cases, post-incubation with probiotics in relation to the time point at which *C. jejuni* was inoculated, led to enhanced adhesion and invasion of both cell types. The probiotic EcN limited the adhesion to and invasion of *C. jejuni* into Caco-2 cells very effectively when it was pre- or coincubated. On CIEC, this effect was weaker and was observed only after preincubation with EcN for *C. jejuni* adhesion and after coincubation for *C. jejuni* invasion. The BS strain reduced *C. jejuni* adhesion and invasion rates on CIECs after pre- and coincubation. While BS had no effect on *C. jejuni* invasion of Caco-2 cells, there was a strong increase in adhesion after probiotic preincubation. Finally, BL led to a decrease in *C. jejuni* invasion after pre- and coincubation of CIECs and Caco-2 cells. In contrast, adhesion was amplified except after coincubation of Caco-2 cells (Experiment 5).

**Fig. 5:**
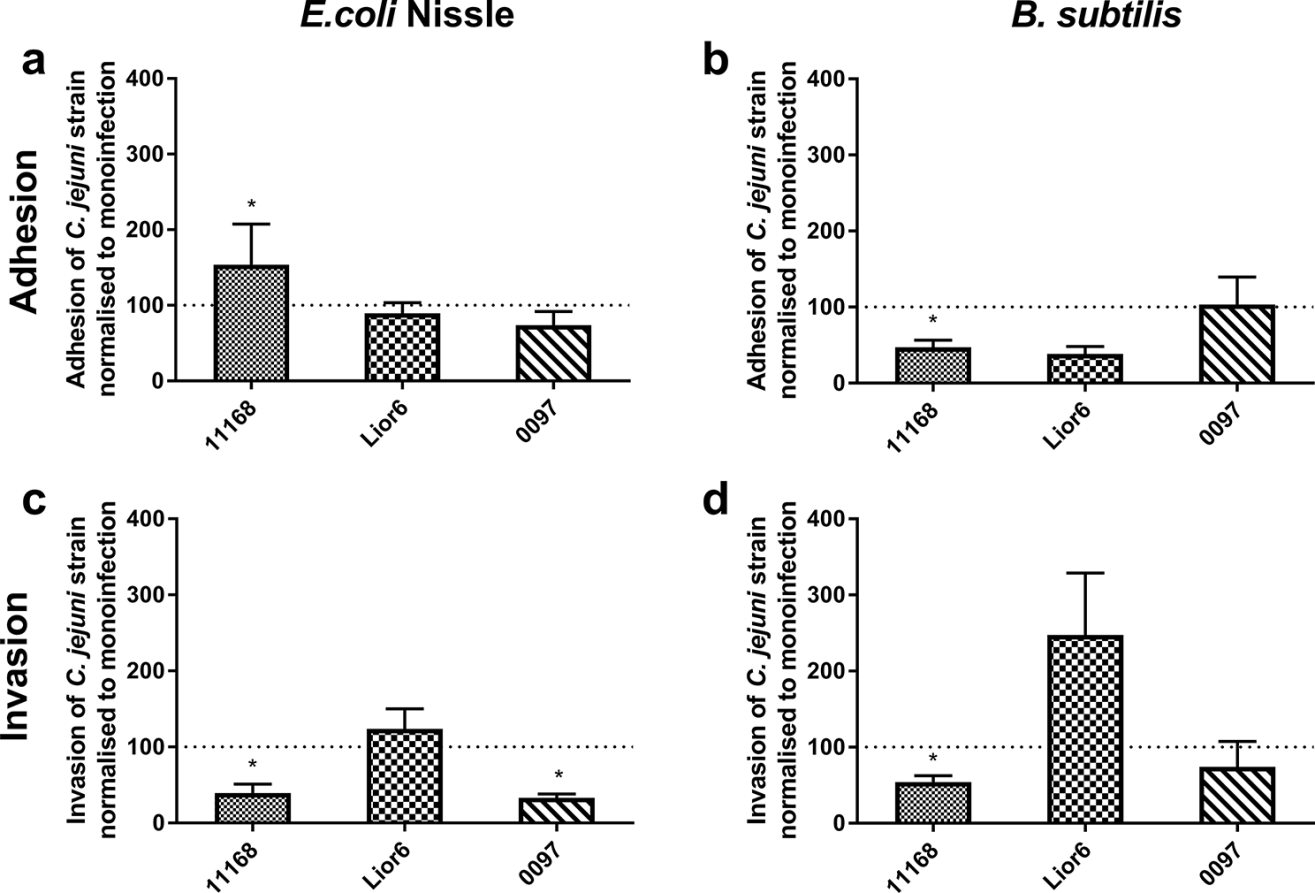
Comparison of possible interference of probiotic candidates on colonization of CIECs by different *C. jejuni* strains. CIECs were inoculated with 10^6^ CFU/ml *C. jejuni* 11168, Lior6 or 0097. The same cells were coincubated with *E. coli* NISSLE (**a**, **c**; inoculation dose 10^6^ CFU/ml) or *B. subtilis* DSM 17299 (**b**, **d**; inoculation dose 10^5^ CFU/ml). The results are presented as the percentage of adherent (**a**-**b**) or invasive (**c**-**d**) cells compared to that of *C. jejuni* monoinfected cells (100%, marked with the dotted line) in the same experiment. The presented data are the means of two independent experiments with pools of different chicken donors performed in triplicate. Error bars indicate the standard error of the mean (SEM). Asterisks indicate significant differences compared to the noninoculated control CIEC at 3 h after *C. jejuni* inoculation (*p* < 0.05; two-sample t test)

In a subsequent experiment (Experiment 6), the probiotic effects on the three different *C. jejuni* strains were compared to determine possible strain-dependent differences resulting from interference with CIECs. Because BL did not significantly inhibit the adhesion of *C. jejuni* 11168 to CIECs, only the effects of EcN and BS were tested. Furthermore, coinoculation was selected because both probiotic effects were observed with *C. jejuni* 11168 following this inoculation schedule. The interfering effects of EcN and BS on *C. jejuni* 11168 were also confirmed in this experiment. Interestingly, neither probiotic candidate had a significant interfering effect on Lior6 adhesion and invasion; however, for *C. jejuni* 0097, EcN clearly reduced invasion, but no significant probiotic effect was observed with BS (*p* > 0.05; Fig. 5).

**Fig. 6:**
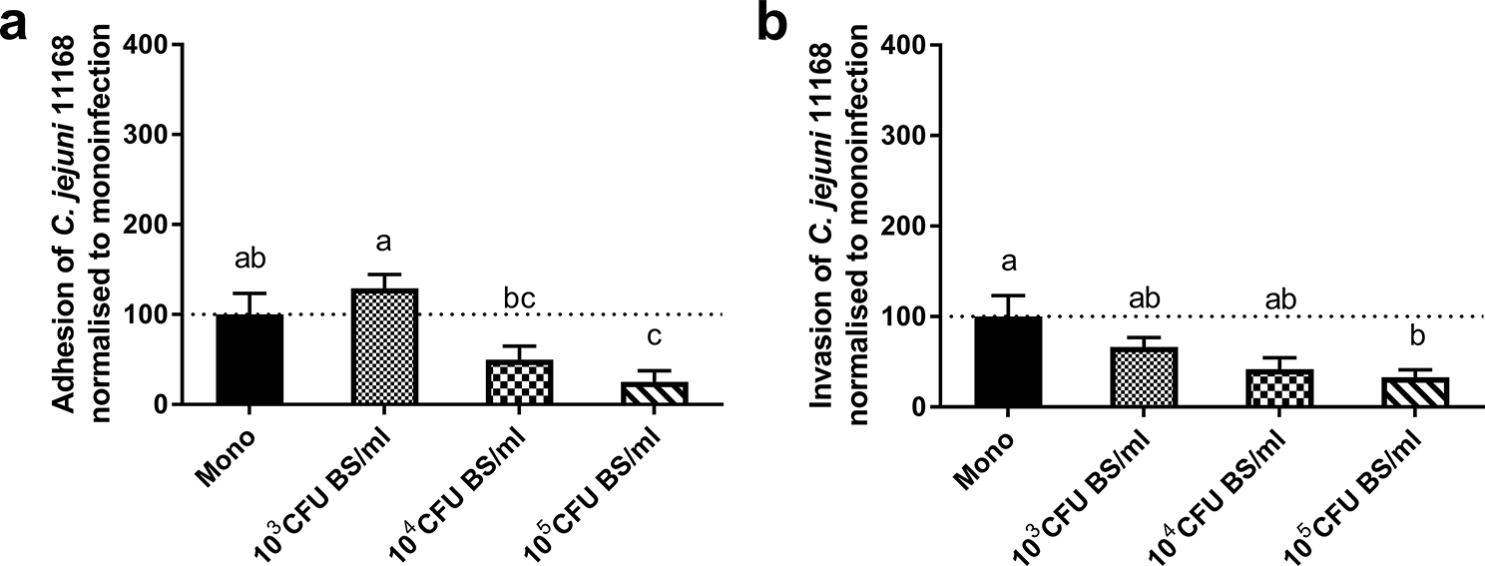
Dose dependency of probiotic effects of *B. subtilis* on *C. jejuni* 11168 adhesion and invasion of CIEC. CIECs were inoculated with 10^6^ CFU/ml *C. jejuni* 11168. The cells were coincubated with 10^5^, 10^4^ or 10^3^ CFU/ml *B. subtilis* DSM 17299. The results are presented as the percentage of adherent (**a**) or invasive (**b**) cells compared to that of *C. jejuni* monoinfected cells (Mono; 100%, marked by the dotted line) in the same experiment. The presented data are the means of two independent experiments with pools of different donor chickens performed in triplicate. Error bars indicate the standard error of the mean (SEM). Letters indicate significant differences between groups. (*p* < 0.05; one-way analysis of variance with Tukey’s HSD All-Pairwise Comparisons Test)

Given that coincubation with BS clearly reduced the adhesion and invasion of *C. jejuni* 11168, we selected this combination to test dose dependency (Experiment 7). Only the highest concentration of 10^5^ CFU/ml BS led to a statistically significant decrease in adhesion and invasion (*p* < 0.05; Fig. 6). Lower concentrations of BS caused lower or no significantly decreased colonization.

## Discussion

Cell lines have shown some differences in their morphology and activity to their primary counterparts of the same origin [25, 26]. In addition a not marginal number of cell lines are contaminated by other cell types or mycoplasma, or the cell lines may be overpassaged, which could lead to doubtful results [27, 28]. Furthermore, available intestinal epithelial cell lines of chickens are derived from the small intestine of chicken embryos [20]. Because the intestinal epithelium undergoes profound changes in morphology and proliferation [29] and because the small intestine is not the main colonization site for *C. jejuni*, these cell lines represent no alternative for our research objective. Therefore, we used primary CIECs to investigate the interaction modes, such as adhesion and invasion, of *C. jejuni* as well as the ability of these interactions to induce proinflammatory cytokines more closely. The effects of three *C. jejuni* strains of different origins and/or colonization properties in vivo [30] were compared, and the dose effects were determined. Furthermore, the possible interfering effects of probiotic candidates were investigated by examining the exclusion, competition and replacement of *C. jejuni*-inoculated CIECs. In selected experiments, Caco-2 cells were also included as a reference to determine possible cell type-associated differences in adhesion and invasion patterns.

We compared the adhesion, invasion and invasion indices of CIECs infected with three different *C. jejuni* strains and evaluated the effects of these strains on the mRNA expression of IL-1β and IL-6 via RT‒PCR. We detected differences in the adhesion and invasion of the tested *C. jejuni* strains. This finding is in accordance with in vitro studies on Caco-2 cells [13, 31] and primary intestinal cells from chicken embryos [16] and mature chickens [32].

In previous in vivo studies with the same *C. jejuni* strains, which were conducted in layer-type birds, we found no clear differences in the cecal colonization of these *C. jejuni* strains at three, seven, 14 and 21 days after inoculation, and only strain 0097 was detected extraintestinal in liver samples [30]. Extraintestinal detection of *C. jejuni* is suggested to be correlated with increased invasiveness in vitro [10, 31]. For that reason, we expected an increased in vitro invasiveness for *C. jejuni* 0097 than for the other tested *C. jejuni* strains, which was confirmed by our experiments.

In the present study, *C. jejuni* 11168 exhibited greater adhesion and particularly invasion to Caco-2 cells than to CIECs. Higher colonization rates in human intestinal epithelial cells in relation to animal intestinal epithelial cells were previously observed [10, 11]. On the other hand, in a study by Byrne et al., only one of six *C. jejuni* isolates showed differences in invasion between primary human and avian intestinal cells [15]. Furthermore, in a model with a permanent embryonic chicken cell line derived from total small intestinal tissue, there were no obvious differences in the colonization of a panel of different *C. jejuni* isolates compared to that of human HT-29 cells [19]. Additionally, comparisons of primary embryonic chicken intestinal cells with permanent human embryonic INT-407 cells revealed similar colonization patterns between the strains [17]. We speculate that these contrasting results could be due to the use of embryonic cells in some studies, while others have used cells from different gut sections of older birds. Therefore, the gut cell location and age of the donor may significantly affect susceptibility to *C. jejuni* infection and invasion. Furthermore, factors such as the developing microbiome in different gut sections may influence the outcome of *C. jejuni* infection [33]. In a previous project, we were able to determine the impact of genotype on the colonization of the chicken gut by *C. jejuni* [34]. In this context, a direct comparison of the colonization of CIECs from layer-type chickens and broiler-type chickens would be very interesting in future studies.

Upregulation of IL-1β and IL-6 mRNA expression was described in the cecum and ileum of broilers after in vivo infection with *C. jejuni* [23, 35]. We investigated the expression of IL-1β and IL-6 mRNAs in the early phase of infection at four and eight hpi with different *C. jejuni* strains. In contrast to the *C. jejuni* strains 11168 and Lior6, only the strain 0097 induced slight but significant upregulation of IL-1β mRNA expression eight hpi and marked upregulation of the IL-6 mRNA expression at four and eight hpi. These results suggest strain-related differences in the stimulation of innate immune responses.

Other studies have also detected variations in the expression patterns of cytokines after inoculation with different *C. jejuni* strains [9, 16]. In an in vivo experiment inoculation with strain Lior6 or 0097 resulted in upregulation of IL-6 mRNA expression, while IL-6 mRNA expression was downregulated after inoculation with strain 11168 [30]. A tissue-specific cytokine response was observed after experimental infection of chickens with *C. jejuni* 81116, in which an early increase in IL-6 mRNA expression was detected in cecal tissue and the spleen, and a delayed IL-1β mRNA expression increase only in the spleen [24].The upregulation of IL-6 mRNA expression at four hours post *C. jejuni* 0097 inoculation (Fig. 5b) suggests a correlation with the invasion index at three hpi (Fig. 3b), which was previously described for IL-8 and *C. jejuni* 81–176 after infection of human embryonic INT407 intestinal cells [36].

Diverse probiotics, such as lactobacilli, were shown to be effective at controlling *C. jejuni* colonization both in vitro and in vivo [37, 38]. None of the probiotic candidates used or their associated soluble factors used in the Agar Well Diffusion Assay in this study had direct antibacterial effects on *C. jejuni*. Therefore, we selected three known probiotics, *E. coli* Nissle 1917 and two Bacillus species, for further studies to investigate their ability to reduce *C. jejuni* adhesion and invasion by coinoculation in vitro. In addition, we investigated the strain- and cell line-specific effects of the strains.

Our study clearly revealed a cell type -possibly species- and *C. jejuni* strain-dependent effect on the probiotics *E. coli* Nissle 1917 and *B. subtilis* DSM 17299. Only EcN clearly reduced the colonization of *C. jejuni* 11168 in Caco-2 cells, while BS only clearly reduced the colonization of CIECs. However, these effects were not reproducible with all tested *C. jejuni* strains. In accordance with our results, a cell type- and pathogen strain-specific effect was also described for probiotic inhibition of *C. jejuni* invasion by *Lactobacillus helveticus* R0052 in human T84 and INT407 cells [14]. Moderately inhibited adhesion and strongly inhibited invasion with variations between the tested *C. jejuni* strains on polarized HT-29 cells caused by preincubation with EcN were shown by Helmy et al. and promoted our results [39]. A reduction in *C. jejuni* colonization in chicken caeca after preincubation with EcN in an in vivo trial by the same study group was also in accordance with our results [40]. Interestingly, *Bacillus licheniformis* DSM 17236 reduced the invasion of *C. jejuni* 11168 into both investigated cell lines, CIECs and Caco-2 cells, while adhesion increased.

The nonpathogenic EcN was shown to reduce the duration of acute enteritis after both bacterial and viral infections [41]. It interfered with various human-derived pathogens, such as *Salmonella* Typhimurium, *Yersinia enterocolitica*, *Shigella flexneri*, *Legionella pneumophila*, and *Listeria monocytogenes, in vitro* in human intestinal cell cultures [42].

*C. jejuni* likely affects tight junctions [43, 44] and invades preferentially and in greater amounts from the basolateral side [45], which could explain the ability of EcN to reduce C. jejuni colonization in Caco-2 cells in our study. EcN was able to restore and protect the barrier function of T84 cells against enteropathogenic *E. coli* [46], and it was even more effective at generating a proinflammatory response from the basolateral side of polarized Caco-2 cells, indicating an improved barrier function in cells with disrupted epithelial barriers [47]. In an animal trial, EcN induced an increase in the amount of the tight junction protein ZO-1 in mice and improved the barrier function of the intestinal epithelium [48]. In other studies, preincubation with EcN led to a reduction in the invasion of *C. jejuni* in human HT-29 cells, maintained epithelial barrier function and modulated the innate immune response [49, 50].

Previously, *B. subtilis* strains were shown to have probiotic effects by inhibiting the growth of various chicken pathogens, including *C. jejuni* [51]. *B. subtilis* BS3 produces two antimicrobial agents that were shown to have growth inhibitory effects on *Helicobacter pylori*, which is closely related to *C. jejuni* [52]. *B. subtilis* DSM 17299 was able to reduce the number of CFU of *Salmonella* Enteritidis in the cecum of chickens by 3 log_10_ [53], but this effect was not reproducible for *C. jejuni in vivo* [54]. The previously observed differences in the probiotic effects of various *B. subtilis* strains also support our studies, suggesting host-, pathogen- and probiotic strain-specific effects. Feed supplemented with *B. subtilis* B10 modulates Toll-like receptor and cytokine expression in the jejunum and ileum of broilers [55]. Modulation of the innate immune system could explain the suppressed *C. jejuni* colonization of CIECs. However, further studies are needed to determine the mechanisms underlying these strain-specific effects, which may be associated with other innate immune parameters not investigated in our experiments.

None of the three probiotics tested had a reducing effect on *C. jejuni* after postincubation. In most cases, adhesion and invasion were two to threefold greater in these groups than in the nontreated controls. This may suggest that only prophylactic and not therapeutic use of these probiotics is suitable for reducing *C. jejuni* colonization.

It is not possible to establish an extremely complex gut ecosystem in a cell culture model. Nevertheless, cell culture systems can provide valuable information about the modes of direct interaction of a single cell type with a specific pathogen. Therefore, we consider CIECs to be a good model for investigating host‒pathogen interactions in more detail in the chicken cecal epithelium and select parameters of interest for further investigation in other in vitro and in vivo models.

Overall, our study provides clear evidence that the type of cell (host origin) and the respective *C. jejuni* strain influence the outcome of the pathogen‒host interaction. In addition, our data provide circumstantial evidence that probiotics may act in a host species-specific manner. The effects may vary not only between pathogens but also between strains in association with the time point of administration. This study paves the way for follow-up investigations because these *C. jejuni*-host interactions and associations among *C. jejuni*, probiotic candidates and hosts need to be investigated further to be able to implement improved control strategies in the field.

## Materials and methods

### Chickens

Specific pathogen-free (SPF) chicken eggs were purchased from VALO BioMedia GmbH (VALO BioMedia GmbH, Osterholz-Scharmbeck, Germany) and incubated until hatching. Chickens were raised in a cage-free aviary system with woodshavings under confined conditions in the facilities of the Clinic of Poultry, University of Veterinary Medicine Hannover. Birds had *ad libitum* access to water and feed („all-mash L“, Deutsche Tiernahrung Cremer GmbH & Co. KG, Düsseldorf, Germany). Between five and twelve weeks posthatch chickens were humanely sacrificed according to the welfare regulations of Lower Saxony, Germany, to collect fresh ceca. Three to nine chickens were sacrificed for each experiment (parts 1–3) to isolate primary chicken intestinal epithelial cells (CIECs) The number of sacrificed chickens was approved and subsequently reported to the authorities according to the German welfare regulations.

### Isolation of primary chicken intestinal epithelial cells (CIECs)

For all experimental parts (parts 1–3) chicken intestinal epithelial cells (CIECs) were isolated as described earlier, with some modifications [32]. Briefly, the ceca of 5-12-week-old SPF-layer chickens were collected aseptically during necropsy, washed in Hank´s Balanced Salt Solution (HBSS), pooled, chopped and digested enzymatically in digestion medium (Dulbecco’s Modified Eagle’s medium (DMEM)/Ham´s F12 (1:1; Biochrom GmbH, Germany, Berlin), 1% fetal bovine serum (FBS; Biochrom GmbH, Germany, Berlin), 50 µg/ml gentamicin (Sigma-Aldrich, USA, St. Louis), 100 U/ml penicillin, 100 µg/ml streptomycin (Biochrom GmbH, Germany, Berlin), 1 U/ml dispase II (Sigma-Aldrich, USA, St. Louis) and 75 U/ml collagenase (Biochrom GmbH, Germany, Berlin)) for 2 hours. Afterwards, single cells and bacteria were removed by using sorbitol gradient centrifugation (DMEM/Ham´s F12 (1:1), 2% d-sorbit (Carl Roth GmbH, Germany, Karlsruhe), 2.5% FBS, 50 µg/ml gentamicin) at 100 × *g* for 3 min at 37 °C. Sorbitol gradient centrifugations were repeated until the supernatant remained clear. The remaining pellet of crypts was resuspended in growth medium (DMEM/Ham´s F12 (1:1), 2.5% FBS, 10 µg/ml insulin (Sigma-Aldrich, USA, St. Louis), 1.4 µg/ml hydrocortisone (Sigma-Aldrich, USA, St. Louis), 5 µg/ml transferrin (Sigma-Aldrich, USA, St. Louis), 1 µg/ml fibronectin (Biochrom GmbH, Germany, Berlin), 100 U/ml penicillin, 100 µg/ml streptomycin; 50 µg/ml gentamicin). Crypt numbers were identified by counting 50 µl of the suspension on Tissue Culture Dishes with Grid (SARSTEDT AG & Co. KG, Nümbrecht, Germany) with an inverted light microscope. The suspension was adjusted to a concentration of 6000 crypts/ml based on preliminary experiments, seeded on collagen-coated 24-well plates (500 µl/well; Greiner Bio-One GmbH, Germany, Frickenhausen) and incubated at 37 °C in a 5% CO_2_ atmosphere. After 24 h, the medium was replaced with fresh growth medium (500 µl/well). Cells were used for further investigations after 24–48 h of incubation, when the cell density of CEIC monolayers was approximately 5 × 10^5^ cells/cm^2^.

### Culture of the permanent human cell line Caco-2

The permanent human colon cell line Caco-2 was cultured as described previously [56]. Caco-2 cells were routinely cultured in growth media (DMEM, 20% FBS, 1% 100 U/ml penicillin, 100 µg/ml streptomycin and 1% nonessential amino acids [all from Biochrom GmbH, Germany, Berlin]) at 37 °C in a 5% CO_2_ atmosphere. The cells were passaged every 2–3 days. For use in the assays, the cells were seeded in 24-well plates and were grown for 2 days before further treatment.

### Bacterial strains

Different bacterial strains were used as probiotic candidates in this study. *Escherichia coli* NISSLE (EcN) was kindly provided by Ardeypharm GmbH, Herdecke, Germany. *Bacillus subtilis* DSM 17299 (BS) and *Bacillus licheniformis* DSM 17236 (BL) was kindly provided by BioChem, Lohne, Germany. *Clostridium butyricum* DSM 10702 (CB), *Enterococcus faecium* DSM 7134 (EF), and *Lactobacillus rhamnosus* DSM 7133 (LR) were kindly provided by Lohmann Animal Health GmbH, Cuxhaven, Germany. Nearly all the probiotic strains were cultured on Columbia Sheep Blood Agar (CSBA) at 37 °C under aerobic conditions for 24 h. *Lactobacillus rhamnosus* DSM 7133 was cultured on MRS agar, and *Clostridium butyricum* DSM 10,702 was cultured on CSBA at 37 °C but under anaerobic conditions for 48 h.

Three different *C. jejuni* strains were used in this study. The *C. jejuni* reference strain NCTC 11168, which was isolated from a human patient, was made available by the Institute for Microbiology and Hygiene at the Charité, Berlin, Germany. *C. jejuni* strain 0097 was kindly provided by the Friedrich-Loeffler-Institute, Jena, Germany, and was isolated from a laying hen. *C. jejuni* strain Lior6 was isolated from a chicken and was part of the strain collection of the Clinic of Poultry, University of Veterinary Medicine Hannover, Germany. All strains were stored in a 10% skim milk suspension at -70 °C. Prior to the experiments, 100 µl of a *C. jejuni* (11168, 0097 or Lior6) suspension in 10% skim milk with 10^6^ CFU/ml was added to 3 ml of sterile Standard-I-Bouillon (Merck, Darmstadt, Germany) supplemented with 1 g/l Deoxycholic acid sodium salt (Carl Roth GmbH + Co. KG, Germany, Karlsruhe), 32 mg/l Cefoperazone and 1 mg/l Amphotericin B (Oxoid, Munich, Germany) and incubated at 37 °C under microaerophilic conditions (CampyGen, Oxoid, Wesel, Germany) for 48 h. After incubation, the suspension was centrifuged for 5 min at 30 × *g*, and the bacteria were resuspended in the required media.

### Experimental procedure

A total of three experimental approaches were conducted to understand the interaction of *C. jejuni* with CIEC and to identify possible modes of interference with probiotic candidates. In part 1 and 3, Caco-2 cells were used as reference cells, and possible differences to CIEC with respect to pathogen‒host interactions were investigated.

#### Part 1: investigations of the interaction between C. jejuni and primary CIECs

In part 1, we investigated the host-pathogen interaction of *C. jejuni* with primary chicken-derived intestinal epithelial cells (CIECs). In the first experiment (Experiment 1), we studied the dose-dependent adhesion and invasion of the *C. jejuni* reference strain 11168 at three different concentrations (10^4^ – 10^6^ CFU/ml). In this experiment, CaCo-2 was used as a reference for comparison. In Experiment 2, we investigated possible strain variations in the adhesion and invasion pattern in CIEC by using three different *C. jejuni* strains (11168, 0097, Lior6; each 10^6^ CFU/ml). In Experiment 3, the proinflammatory host response of CIECs was further investigated by measuring the expression patterns of the selected cytokines IL-1β and IL-6 after four and eight hours of incubation with the three selected *C. jejuni* strains (11168, 0097, and Lior6; each with 10^6^ CFU/ml).

#### Part 2: investigation of the soluble factor-mediated probiotic effects of six selected probiotic candidates on C. jejuni

Six different probiotic candidates (EcN, BS, BL, CB, EF and LR) were investigated for interference with possible soluble factors, which may be released during propagation in growth media during the replication of *C. jejuni* strains (11168, 0097, Lior6). The Agar Well Diffusion Assay was used in this experiment (Experiment 4).

#### Part 3: investigations of the indirect probiotic effects of three selected probiotic candidates

We investigated the ability of three selected probiotic candidates (EcN, BS and BL) to reduce the colonization of *C. jejuni* 11168 in CIECs via interference assays. Caco-2 cells were used as a reference (Experiments 5 + 6). In addition, to identify possible *C. jejuni* strain variations, EcN and BS were selected and tested for possible interfering effects on three *C. jejuni* strains, 11168, 0097 and Lior6 (10^6^ CFU/ml each), in an interference assay on CIECs (Experiment 7). BS was further selected to identify possible dose variations at 10^3^-10^5^ CFU/ml upon interference with 10^6^ CFU/ml *C. jejuni* 11,168 (Experiment 8).

Table 1 provides an overview of the type of cells used and the number of trials and replicates per experiment.

### Adherence and invasion assay

The adherence and invasion of *C. jejuni* were investigated by using the Gentamicin Protection Assay [32, 57]. Wells with CIECs or Caco2 cells were washed twice with DMEM and covered with conservation media (DMEM/Ham´s F12 (1:1), 2.5% FBS, 10 µg/ml insulin), after which confluence was evaluated. Only wells with a confluence above 75% were used for further investigations. Bacterial suspensions of *C. jejuni* were adjusted to the required concentration by the use of the McFarland turbidity standard following standard procedures, and CFU were confirmed retrospectively by 10-fold serial dilution and plating [35]. The cells were covered with 500 µl of bacterial suspension and incubated for 3 h at 37 °C in a 5% CO_2_ atmosphere. Afterwards, the wells were washed three times, after which the cells were lysed with 500 µl of 0.5% Triton X-100, and serial dilutions of the lysates were prepared and subsequently plated on *Campylobacter*-selective charcoal-cefoperazone-deoxycholate agar (Campylobacter CCDA Selective Medium; Oxoid, Wesel, Germany) to determine the presence of adherent *C. jejuni*. For determination of invading cells, the wells were washed three times with DMEM and incubated with conservation media supplemented with 100 µg/ml gentamicin. After 1 h, the wells were washed three times with DMEM and lysed with 500 µl of 0.5% Triton-X 100. The number of invaded *C. jejuni* cells was determined after 10-fold serial dilution of the lysed samples and plating on CCDA plates. The CCDA plates were incubated for 48 h at 37 °C under microaerophilic conditions prior to counting. The enumeration of adherent *C. jejuni* included the total number of *C. jejuni* associated with cells prior to lysis (for extra and intracellular bacteria) [57]. The results are expressed as the percentage of CFU of *C. jejuni* in the inoculum that adhered or were internalized (relative adhesion and invasion) or as the percentage of total cell-associated *C. jejuni* that was internalized (InvasionIndex [57]).

### Agar well diffusion assay

The Agar Well Diffusion Assay was conducted as described previously by Campana et al. with slight modifications [58]. One colony of *Escherichia coli* NISSLE, *Clostridium butyricum* DSM 10702, *Bacillus subtilis* DSM 17299, *Bacillus licheniformis* DSM 17236, *Enterococcus faecium* DSM 7134 or *Lactobacillus rhamnosus* DSM 7133 was suspended in five ml of sterile Standard I Bouillon and incubated at 37 °C under aerobic conditions. CB was incubated under anaerobic conditions. Noninoculated standard I Bouillon plants were treated in the same way and used as a negative control. After 48 h of incubation, the bacterial suspensions were centrifuged at 30 × *g* for 10 min. The supernatants were filtered (VWR Syringe Filters, VWR International, Radnor, USA; 0.22 mm pore size) to remove the remaining bacteria. Samples of all cell-free supernatants (CFS) were spread out on Columbia Sheep Blood Agar and incubated under aerobic, anaerobic and microaerophilic conditions at 37 °C for 48 h to confirm the absence of any remaining bacteria. The supernatants were stored at -20 °C until use.

Standard I Bouillon (Merck KGaA, Germany, Darmstadt) with 1% Agar Agar (Carl Roth GmbH + Co. KG, Germany, Karlsruhe) was autoclaved and cooled. At a temperature of 45 °C, 100 µl of a *C. jejuni* (11168, 0097 or Lior6) suspension in 10% skim milk with 10^6^ CFU/ml was added to 200 ml of the bouillon with Agar Agar. Subsequently, 22 ml of this suspension was added to each Petri dish, and after solidifying, five wells each five mm in diameter were punched into the agar under sterile conditions. Three wells were filled with 48 µl of CSF of the same source, one well with the negative control and one well with a Gentamicin solution (positive control; 10 mg/ml; Sigma-Aldrich, USA, St. Louis). Plates were incubated for 48 h at 37 °C under microaerophilic conditions (CampyGen, Oxoid, Wesel, Germany). Antimicrobial activity led to a clear inhibition zone around the subsequent well, while the remaining agar got turbid due to *C. jejuni* replication.

### Interference assay

One possible mechanism of probiotic action is competitive exclusion. According to this principle, one bacterial species competes more vigorously for receptor sites in the intestinal tract than does another species. Additionally, other mechanisms, such as competition for nutrients, creation of a hostile microecology or secretion of antimicrobial substances, have been described [59]. If a probiotic species has the ability to occupy a particular ecological niche before the pathogen, this process is termed exclusion. An effect based on simultaneous colonization is named competition, and displacement describes a probiotic effect, which leads to the reduction of an already established colonization of another bacterial species. The interference assay was conducted as described previously [14, 56, 58], with slight modifications, to investigate Competitive Exclusion effects. CIECs and Caco-2 cells were prepared and treated in the same way as for the adherence and invasion assays. The bacterial concentrations were adjusted to 10^6^ CFU/ml for *C. jejuni*, EcN and BL and 10^5^ CFU/ml for BS in the final mixture of bacteria in conservation media. For the investigation.

1. After exclusion, the cells were washed with DMEM and pretreated with one of the probiotic candidates. After 1 h of preincubation, the *C. jejuni* strain was added.
2. After competition, the cells were incubated with a mixture of one *C. jejuni* strain and one of the probiotic candidates.
3. After displacement, the cells were preincubated for 1 h with the *C. jejuni* strain prior to the addition of one probiotic candidates.

For adherence and invasion assays, the incubation time was stopped three hours after the addition of *C. jejuni* to the cells, and the cells were further processed for adherence and invasion analysis as described above.

### qRT‒PCR detection of the mRNA expression of selected cytokines

Four and eight hours after inoculation with one of the *C. jejuni* strains (11168, 0097, Lior6; each 10^6^ CFU/ml), the CIECs were washed and detached with 250 µl of trypsin/EDTA (0.05%/0.02%; Biochrom GmbH, Germany, Berlin). After detachment, the cells were stored at -80 °C until RNA isolation.

Total RNA was extracted from cell samples by using the MasterPure RNA Purification Kit (Epicentre, USA) according to the manufacturer´s instructions. The isolated RNA was stored at -80 °C until qRT‒PCR analysis.

qRT‒PCR was performed by using a Stratagene MX 3005P RT-qPCR cycler (Stratagene, USA) and an AgPath-ID One-Step RT‒PCR Kit (Applied Biosystems, USA) according to the manufacturer´s instructions as described previously [33]. The primers and probes used for the detection of the mRNA expression of IL-1β and IL-6 as well as the constantly expressed housekeeping gene 28 S were previously published [33, 60, 61]. Three µl of total RNA in 25 µl of reaction mix were used with the following cycle profile: one cycle at 45 °C for 10 min and 95 °C for 10 min and 40 cycles of 95 °C for 15 s and 57 °C for 45 s. The cycle threshold (C_t_) values of the expressed mRNAs of the investigated genes were normalized against those of the expressed housekeeping gene 28 S rRNA of the same sample (ΔC_t_) as described by Powell et al. [62]. The overall 28 S rRNA expression was comparable between samples independent of the treatment. The ΔC_t_ values of the samples are presented as fold changes and were related to the ΔC_t_ values from negative control groups at the same sampling time point.

### Statistical analysis

Statistical analyses were performed with Statistix version 10.0 (Analytical Software, Tallahassee, FL, USA). *p* < 0.05 was considered to indicate statistical significance. In Experiments 1 and 5, two sample t tests and Wilcoxon rank sum tests were used; in Experiments 2 and 7, one-way analysis of variance was performed with the Tukey HSD All-Pairwise Comparisons Test. In Experiment 3, one-way analysis of variance with Tukey’s honestly significant difference (HSD) All-Pairwise Comparisons Test and Two-sample T test were applied. Experiment 6 was statistically verified by a two-sample t test. For the number of trials and replicates, see Table 1.

**Table 1:** Number of sacrified chickens, trials and total replications

Experimental Part	Experiment	Description	Used cells		Sacrificed Chickens^1^	Number of trials	Number of replicates^2^
			CIEC	Caco-2			
1	1	Colonisation pattern after infection with different concentrations of *C. jejuni* 11168	+	+	9	2-3	6-9
1	2	Colonisation pattern after infection of CIEC with three different *C. jejuni* strains	+		8	3	9
1	3	IL-1β and IL-6 mRNA expression in CIEC after *C. jejuni* infection	+		3	1	5-7
2	4	Agar Well Diffusion Assay			0	1	3
3	5	Influence of incubation time point of probiotic candidates on colonisation of CIEC by C. jejuni 11168	+		5	2	6
3	6	Influence of incubation time point of probiotic candidates on colonisation of Caco2 cells by C. jejuni 11168		+	0	2	6
3	7	Influence of probiotic candidates on colonisation of CIEC by different *C. jejuni* strains	+		6	2	6
3	8	Influence of probiotic concentration on colonisation of CIEC by *C. jejuni* 11168	+		7	2	6

^1^: Number of total sacrificed SPF-chickens per experiment used to prepare CIEC. Isolated cells were pooled within a trial.

^2^: Number of total replicates per treatment group and time point investigated.

## Data Availability

The raw data will be made available upon request.

## Abbreviations

BL: Bacillus licheniformis DSM 17236
BS: Bacillus subtilis DSM 17299
C. jejuni: Campylobacter jejuni
CCDA: charcoal-cefoperazone-deoxycholate agar
CIEC: chicken intestinal epithelial cells
CB: Clostridium butyricum DSM 10702
CFU: colony forming units
CFS: cell-free supernatants
CSBA: Columbia Sheep Blood Agar
FBS: fetal bovine serum
EF: Enterococcus faecium DSM 7134
LR: Lactobacillus rhamnosus DSM 7133
hpi: hours post inoculation
IL: interleukin
